# New grading scale based on early factors for predicting delayed cerebral ischemia in patients with aneurysmal subarachnoid hemorrhage: a multicenter retrospective study

**DOI:** 10.3389/fneur.2024.1393733

**Published:** 2024-05-31

**Authors:** Shishi Chen, Hongxiang Jiang, Peidong He, Xiangjun Tang, Qianxue Chen

**Affiliations:** ^1^Department of Neurosurgery, Renmin Hospital of Wuhan University, Wuhan, China; ^2^Department of Neurosurgery, Jingzhou Central Hospital, Jingzhou, China; ^3^Hubei Provincial Clinical Research Center for Umbilical Cord Blood Hematopoietic Stem Cells, Taihe Hospital, Hubei University of Medicine, Shiyan, Hubei, China; ^4^First School of Clinical Medicine of Wuhan University, Wuhan, China; ^5^Department of Neurosurgery, Taihe Hospital, Hubei University of Medicine, Shiyan, Hubei, China

**Keywords:** subarachnoid hemorrhage, delayed cerebral ischemia, prediction model, grading scale, nomogram validation

## Abstract

Delayed cerebral ischemia (DCI) could lead to poor clinical outcome(s). The aim of the present study was to establish and validate a predictive model for DCI after aneurysmal subarachnoid hemorrhage (aSAH) based on clinical data. Data from a series of 217 consecutive patients with aSAH were reviewed and analyzed. Related risk factors within 72 h after aSAH were analyzed depending on whether DCI recurred. Least absolute shrinkage and selection operator (LASSO) analysis was performed to reduce data dimensions and screen for optimal predictors. Multivariable logistic regression was used to establish a predictive model and construct a nomogram. Receiver operating characteristic (ROC) and calibration curves were generated to assess the discriminative ability and goodness of fit of the model. Decision curve analysis was applied to evaluated the clinical applicability of the predictive model. LASSO regression identified 4 independent predictors, including Subarachnoid Hemorrhage Early Brain Edema Score (i.e., “SEBES”), World Federation of Neurosurgical Societies scale score (i.e., “WFNS”), modified Fisher Scale score, and intraventricular hemorrhage (IVH), which were incorporated into logistic regression to develop a nomogram. After verification, the area under the ROC curve for the model was 0.860. The calibration curve indicated that the predictive probability of the new model was in good agreement with the actual probability, and decision curve analysis demonstrated the clinical applicability of the model within a specified range. The prediction model could precisely calculate the probability of DCI after aSAH, and may contribute to better clinical decision-making and personalized treatment to achieve better outcomes.

## 1 Introduction

Aneurysmal subarachnoid hemorrhage (aSAH) is regarded to be a critical type of hemorrhagic stroke, carrying a risk for morbidity and even mortality (1). Previous studies have indicated that delayed cerebral ischemia (DCI) plays an important role in the progress and poor prognosis of aSAH (2). The occurrence of DCI can be affected by many variable factors, including demographics (sex, age, and medical and personal history), radiological changes at admission, and clinical status (3). Previous research has explored several hierarchical systems based on radiological or clinical factors to predict outcome(s) and guide the treatment of DCI (4).

Limitations associated with radiological scores, however, may underestimate the significance of individual clinical signs. As such, several comprehensive evaluation systems have been introduced, such as the HAIR score [Hunt and Hess grade (HH), age, intraventricular hemorrhage (IVH), and rebleeding within 24 h] and VASOGRADE (VG) scale, to predict the prognosis of patients with aSAH (5). Several predictive grading systems have been proposed to explore the occurrence of DCI, and to personalize management and treatment after diagnosis of aSAH (6). These prognostic scores have included radiological, clinical, and composite scores. Nevertheless, none of these grading systems are able to account for the severity and number of bleeds (4). In fact, clinical scores chiefly applied to neurological deficits and mental status, which reflect changes and severity related to brain injury after aSAH (7). The World Federation of Neurological Society (WFNS) scale has been the most popular clinical scoring method in the practice of treating aSAH (4); however, adverse effects should not be ignored. It is noteworthy that by analyzing the location and thickness of subarachnoid blood present in imaging data, the Fisher Scale (FS) and modified FS (mFS) can be used to predict DCI and the occurrence of cerebral vasospasm (8). Currently, a new scoring system, known as the Subarachnoid Hemorrhage Early Cerebral Edema Score (SEBES), quantifies the degree of cerebral edema to predict the possibility of DCI. However, further research is required to explore the effectiveness and accuracy of this score (9). These radiological scales underestimate the significance of patient clinical features. Therefore, some composite grading systems, such as the HAIR score and VG scale, have been promoted to predict the recurrence of DCI (5), although the validity and accuracy of these scales remain controversial (10).

It is, therefore, imperative to develop a more comprehensive and systematic grading score that also incorporates radiological and clinical factors and brain changes to predict DCI in a timely manner. Due to the multifactorial complexity of the development of aSAH, a novel risk score should combine risk factors with risk stratification as early as 72 h after aSAH. In clinical practice, a precise and accurate grading system can provide timely evidence to prevent DCI and improve the prognosis of patients who experience aSAH.

## 2 Materials and methods

### 2.1 Study population

This retrospective observational cohort study included 347 patients with SAH who were admitted to the department of neurosurgery between 1 January 2019 and 1 September 2020 at Department of Neurosurgery, Jingzhou Central Hospital and Renmin Hospital of Wuhan University. SAH was assessed by initial lumbar puncture or computed tomography (CT) angiography according to current guidelines. Patients with arteriovenous malformation ruptures and negative angiograms were excluded. Exclusion criteria were as follows: related history of cerebral injury (such as cerebral hemorrhage and stroke, which reflect chronic changes on CT); SAH caused by trauma or questionable trauma; complicated with malignant tumor, serious coagulation disorders, hypertension, and uncontrollable heart disease, which may confound clinical judgment; CT performed >72 h after the initial onset of SAH; and unavailability of initial CT data. The study protocols were approved by the ethics committee of Clinical Research, Renmin Hospital of Wuhan University (WDRY2022-KS003).

### 2.2 Variables

Relevant clinical and radiological data and demographic information of the enrolled patients were obtained from the electronic medical record system. Clinical variables included HH scale and WFNS grade. Unfavorable clinical status was defined as high HH (4–5) and WFNS (4–5). Additionally, radiological factors included intraparenchymal hematoma and IVH identified on CT, and SEBES and mFS scale scores. Serious cerebral edema was defined as a high SEBES (3–4), and high mFS (3–4) defined a large amount of bleeding. Demographic information included sex, age, alcohol consumption, smoking status, diabetes, hypertension, hyperlipidemia, and use of anticoagulants.

### 2.3 Outcomes

The end of follow up was defined as the appearance of DCI, which included delayed cerebral infarction and/or clinical vasospasm. New cerebral infarction on brain CT that was not visible on brain CT, which excluded infarctions that occurred around the aneurysm ≤ 2 days after endovascular treatment or aneurysm clipping, was defined as DCI. Clinical change [Glascow Coma Scale (GCS) score decreased by ≥2 points, or occurrence of new dyskinesia, which excluded other etiologies] was regarded as cerebral vasospasm. All patients underwent either aneurysm clipping or coil embolization. Routine postoperative routine therapies included analgesics, hemostasis, nimodipine, and anti-inflammatory drugs for anti-vasospasm. Other complications, such as hydrocephalus, seizures, and rebleeding, were recorded and managed as appropriate. All radiological data were retrospectively and independently assessed by 2 senior neurosurgeons in the authors’ center, who were blinded to patient information.

### 2.4 Statistical analysis

Statistical analysis was performed using R software (R Foundation for Statistical Computing, Vienna Austria)^1^ or SPSS version 22.0 (IBM Corporation, Armonk, NY, United States); differences with *p* < 0.05 were considered to be statistically significant. LASSO regression was used to analyze highly dimensional data and select the most significant prognostic variables. The variables were subsequently incorporated into multivariable logistic regression analysis through the integration of independent factors to establish the nodal model. Calibration and discrimination capacities assessed the capabilities of the nomogram model. Discriminatory performance was evaluated using ROC curve and area under the ROC curve (AUC) analyses, and 1,000 bootstrap errors were calculated to correct the C-index. The Hosmer–Lemeshow test, calibration curve, and Brier score were performed to evaluate calibration ability.

Predictors identified in multivariable logistic regression were used to develop a risk stratification score designed to explore the development of DCI. According to the method of rounding to the nearest integer and ratio corresponding to B to minimum B (Bx/Bmin), all predictors were assigned an associated risk score, and the selected predictor was graded new score again. ROC curve analysis was used to test the discriminative ability of the new score, and compared with the HH, mFS, SEBES, WFNS, and other scoring systems to predict DCI. Results of the Hosmer–Lemeshow test and *p* > 0.05 defined exceptional calibration.

## 3 Results

### 3.1 Patient characteristics

**Figure 1:**
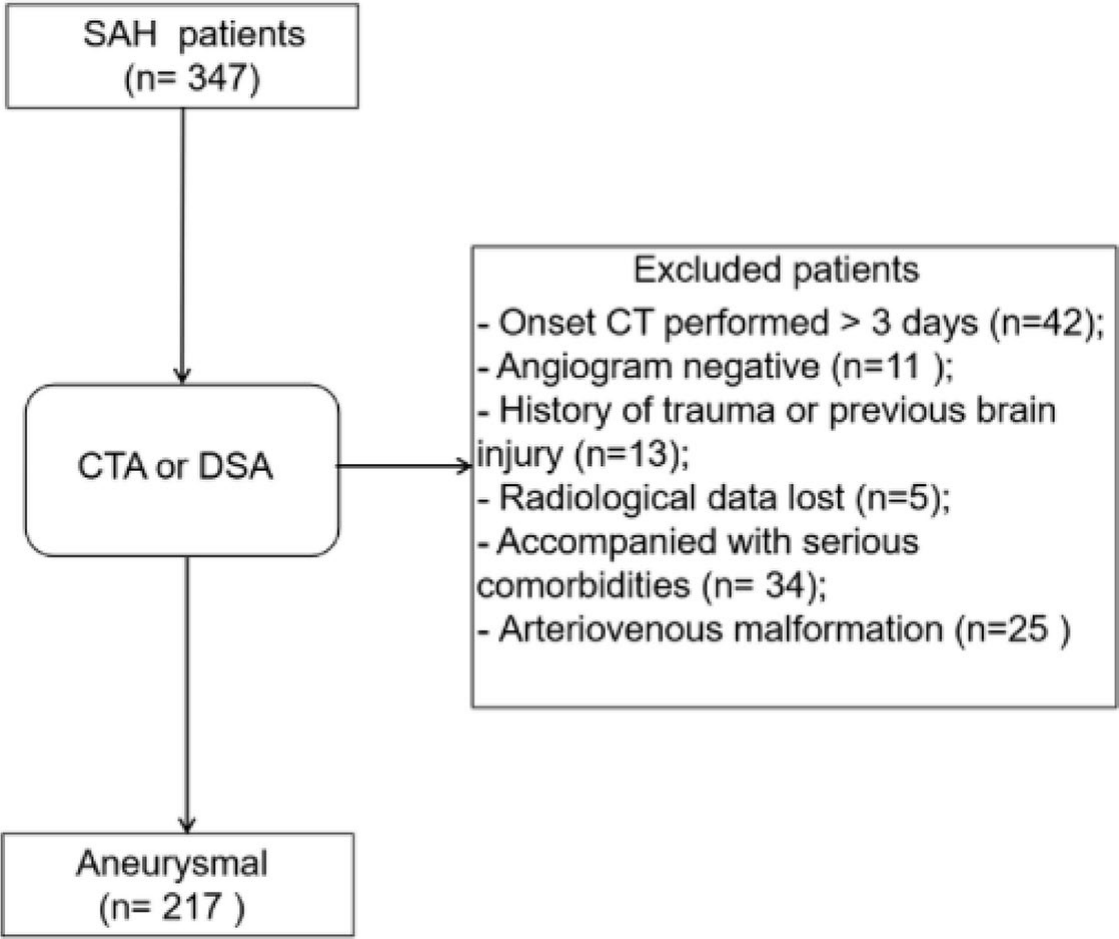
Flow-diagram illustrating the patient cohort with aneurysmal subarachnoid hemorrhage (aSAH). Data from 347 patients with SAH, diagnosed between 2019 and 2020, were retrospectively reviewed, of whom 217 were included after screening.

Patients with the following characteristics were excluded before analysis: negative angiogram (*n* = 11); diagnosed with arteriovenous malformation (*n* = 25); history of trauma or brain injuries (*n* = 13); accompanying critical comorbidities (*n* = 34); received treatment(s) > 72 h after occurrence of symptoms (*n* = 42); and missing radiological data (*n* = 5). Ultimately, data from 217 patients with aSAH were enrolled in the study (Figure 1). The incidence of DCI was 27.6% (60/217), and patients with DCI tended to exhibit higher mFS (*p* < 0.001), SEBES (*p* = 0.001), HH (*p* < 0.001), WFNS (*p* < 0.001) scores, and IVH (*p* < 0.001). Patients with DCI were likely to exhibit intraparenchymal hematoma on CT imaging (*p* < 0.001). Interestingly, differences in the following variables were statistically insignificant: age; sex; history of smoking or alcohol consumption; and history of hypertension, hyperlipidemia, diabetes, and/or antiplatelet or anticoagulant therapies. Additionally, there was no statistical difference between patients with multiple aneurysms or aneurysm size between the DCI and non-DCI groups (Table 1).

**Table 1:** Univariate analysis of characteristics of SAH Patients.

Variable	Non-DCI (*n* = 157)	DCI (*n* = 60)	*P*-value
**Risk factors**			
Gender, male	182 (36.0)	82 (41.4)	0.150
**Age**			
Mean	55.6 ± 11.3	57.0 ± 11.1	0.139
>50	324 (64.0)	137 (69.9)	0.142
>60	171 (33.8)	81 (41.3)	0.062
>70	50 (9.9)	16 (8.2)	0.484
Smoker	111 (21.9)	52 (26.5)	0.196
Drinker	138 (27.3)	64 (32.7)	0.158
Hypertension	192 (37.9)	79 (40.3)	0.564
Hyperlipidemia	180 (35.6)	67 (34.2)	0.729
Diabetes	20 (4.0)	9 (4.6)	0.703
Previous heart disease	4 (0.8)	3 (1.5)	0.406
Antiplatelet or anticoagulant	25 (4.9)	11 (5.6)	0.718
**Clinical variable**			
WFNS 4-5	61 (12.1)	101 (51.5)	<0.001
H-H 4-5	53 (10.5)	74 (37.8)	<0.001
**Radiological variable**			
mFS 3-4	274 (54.2)	170 (86.7)	<0.001
SEBES 3-4	217 (42.9)	134 (68.4)	0.001
IVH	140 (27.7)	122 (62.2)	<0.001
Hematoma	45 (8.9)	55 (28.1)	<0.001
**Aneurysm**			
Anterior circulation	290 (7.3)	128 (65.3)	0.011
Multiple aneurysms	25 (4.9)	16 (8.2)	0.102
Aneurysm size^b^	4.5 ± 2.1	4.6 ± 3.0	0.538

### 3.2 Variable selection

**Figure 2:**
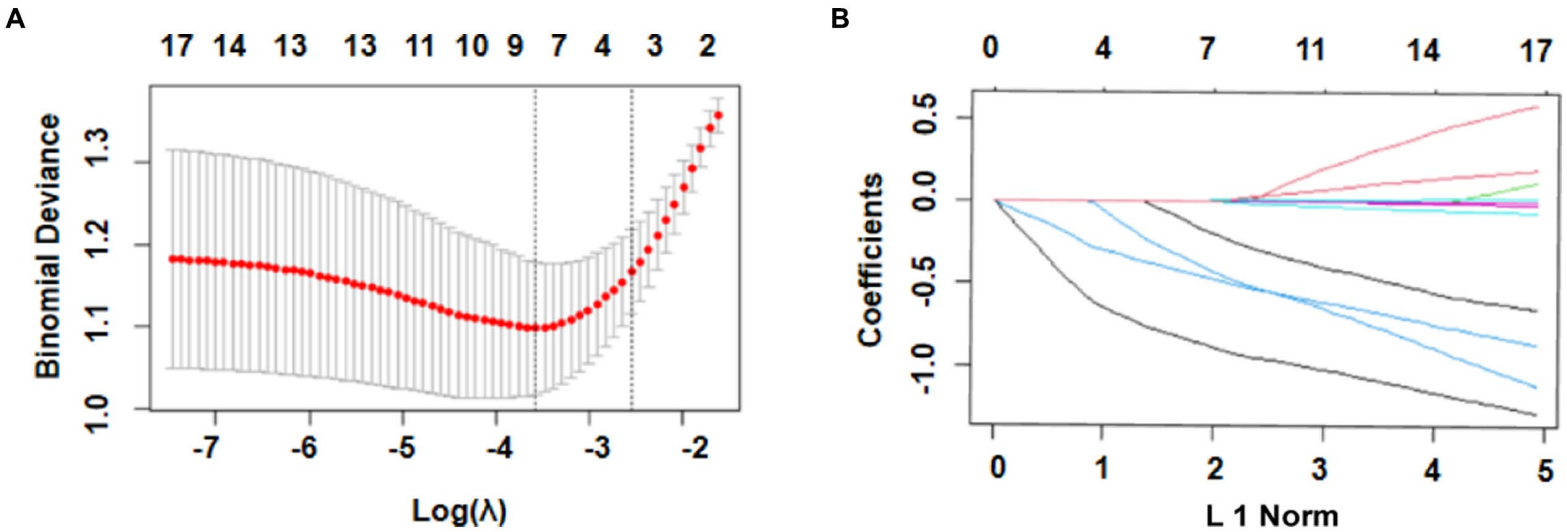
Selection of prognostic variables using least absolute shrinkage and selection operator (LASSO) regression analysis. **(A)** Selection of optimal parameters (lambda) using 5-fold cross-validation. The left and right dotted vertical lines, respectively, represent the optimal lambda values when using the minimum error criterion and one standard error (1–SE) of the minimum criterion; **(B)** The vertical line was plotted with log(λ) = −1.986. Four factors with non-zero coefficients were ultimately selected.

The most significant variables were identified using LASSO regression. As shown in Figure 2A, 17 variables were reduced to 4 variables using a 1 – standard error (SE) criterion. Then, with log(λ) = −2.642, the 4 factors with non-zero coefficients were selected using 5-fold cross-validation to prevent overfitting (Figure 2B). After adjustment in multivariable logistic regression, high WFNS (95% confidence interval [CI] 2.627–6.266; *p* < 0.001), high mFS (95% CI 1.589–4.331; *p* = 0.002), high SEBES (95% CI 1.028–2.305; *p* = 0.036) scores, and IVH (95% CI 1.284–2.906; *p* = 0.003) were independent prognostic factors. Based on the regression coefficient, the correlation score for each predictor was named (Table 2). The novel score, called the “EDCI score,” could be applied to predict the incidence of DCI.

**Table 2:** Multivariate risk stratification score to predict DCI after SAH.

Variables	B (SE)	OR	95%CI	*P*-value	Risk score
High WFNS	1.401 (0.222)	4.057	2.627–6.266	<0.001	3
High mFS	0.964 (0.256)	2.623	1.589–4.331	0.002	2
High SEBES	0.431 (0.206)	1.539	1.028–2.305	0.036	1
IVN	0.658 (0.208)	1.932	1.284–2.906	0.003	1
Intercept	−2.587 (0.229)			<0.001	

### 3.3 Development of the prognostic model

**Figure 3:**
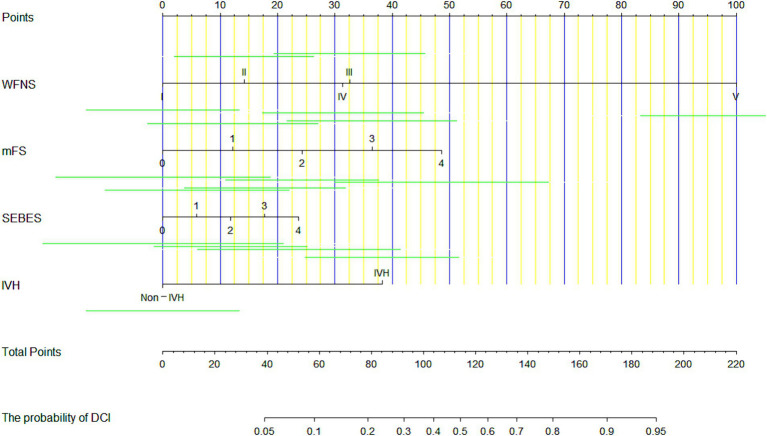
A nomogram for predicting the occurrence of delayed cerebral ischemia (DCI) in patients after aneurysmal subarachnoid hemorrhage (aSAH), based on 4 predictors.

Each independent factor was applied to build a nomogram to predict the occurrence of DCI within 72 h after aSAH (Figure 3). According to the model, all prognostic factors were projected upward to a specific point in the nomogram. The sum of these points from the 4 factors was transferred to an independent poor prognosis score, with a higher score representing a greater tendency toward an unfavorable prognosis.

### 3.4 Model performance

**Figure 4:**
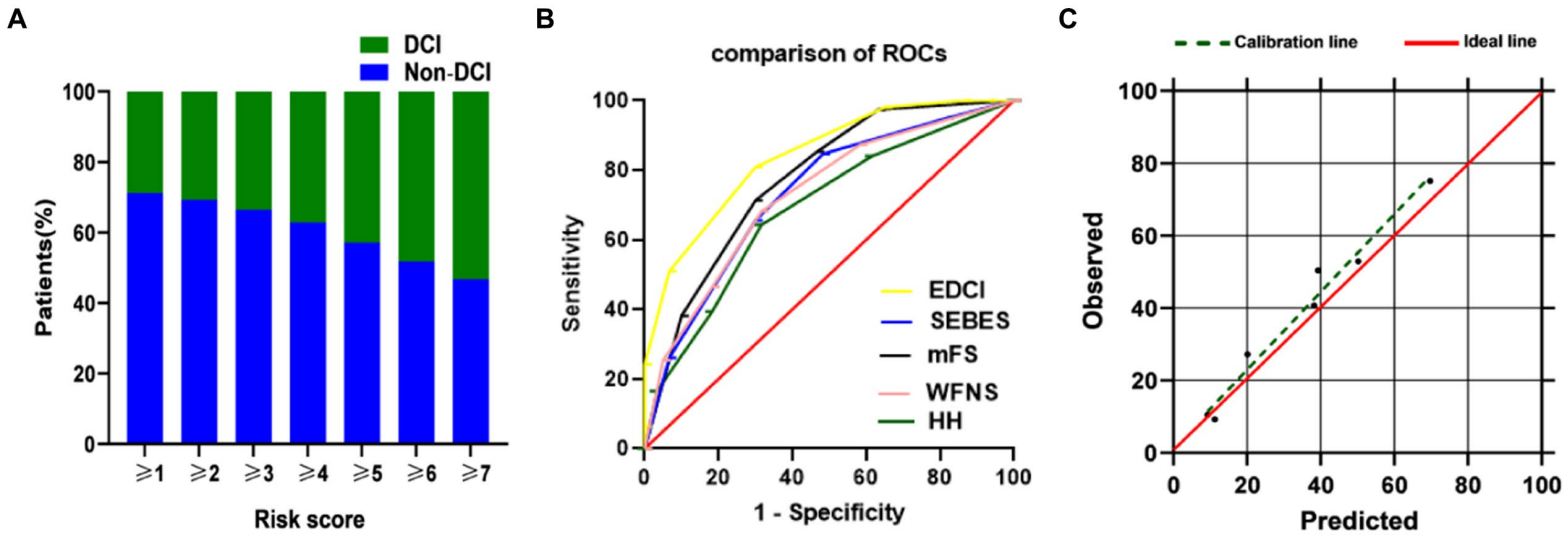
**(A)** DCI rate based on EDCI score. Distribution of the EDCI score and corresponding observed DCI rate; **(B)** ROC of EDCI score and other grading systems. EDCI score keeps a highest AUC (AUC = 0.840, 95%CI = 0.783–0.896) among these grading systems. *p* value was <0.0001 compared to each score; **(C)** Calibration plot for predicted vs. observed DCI for the risk EDCI score. AUC, area under receiver operating characteristics curve; CI, confidence interval; DCI, delayed cerebral ischemia; HH, Hunt-Hess; mFS, modified Fisher Scale; ROC, receiver operating characteristics curve; SEBES, Subarachnoid Hemorrhage Early Brain Edema Score; WFNS, World Federation of Neurosurgical Societies.

The new EDCI score is presented in Figure 4A. The increase in DCI rate was correlated with an increase in score. Furthermore, the discriminative ability of the new EDCI risk score was excellent, with an AUC of 0.840 (95% CI 0.783–0.896; Figure 4B). Its discriminative ability was definitely higher than other radiological and clinical scores. The new EDCI score, compared with the other grading systems, yielded the following AUCs: AUC_WFNS_ 0.774; AUC_HH_ 0.732; AUC_SEBES_ 0.689; and AUC_mFS_ 0.728. The calibration ability was also perfect according to the Hosmer–Lemeshow test (*p* > 0.05; Figure 4C).

### 3.5 Nomogram validation

**Figure 5:**
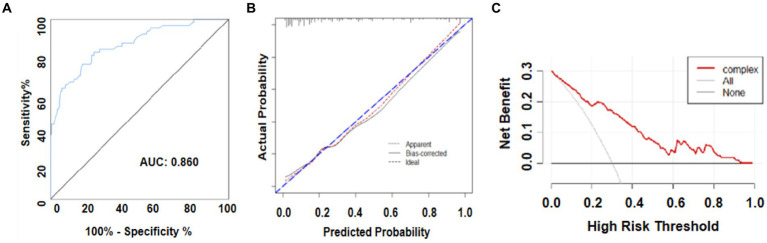
**(A)** Receiver operating characteristic (ROC) curve and area under the ROC curve (AUC) analyses for the predictive model. **(B)** Calibration curve for the predictive model. **(C)** Decision analysis (DCA) curve.

The primary AUC was 0.860 (95% CI 0.802–0.917), indicating that the model demonstrated exemplary discriminative ability. For a single independent predictor, the nomogram model could better predict the incidence of DCI (Figure 5A). The calibration capacity was also internally validated: the Hosmer–Lemeshow *p*-value of 0.43 in the training cohort indicated good fitting of the nomogram. Additionally, a Brier score of 0.11 and a calibration curve with 1,000 bootstrap resamples demonstrated that the model demonstrated good calibration ability (Figure 5B), demonstrating no significant deviation between the predicted probabilities and the actual. Additionally, decision curve analysis (DCA) was performed to assess the clinical application of the prognostic nomogram. DCA revealed a superior overall net benefit for a threshold probability of 0–1 (Figure 5C).

## 4 Discussion

We constructed and verified a dynamic nomogram model to predict the occurrence of DCI by analyzing data obtained early, more specifically, within 3 days after aSAH. This new nomogram consists of four predictors, mFS, SEBES, and WFNS scores, and IVH, and is named the “EDCI score.” The EDCI score combines both radiological and clinical factors, reflecting early brain changes after aSAH, and avoids the one-sidedness of a single scoring system. The larger AUC meant that EDCI functioned better in predicting DCI than other scores. Moreover, a web-based dynamic nomogram is definitely intuitive and easily evaluated by neurosurgeons, and is a significantly more practical and accurate tool compared with conventional predictive models.

Current research investigating cerebral injury in the first 3 days after bleeding is of great importance to the progress of outcomes and complications after aSAH (9). SAH scoring systems can be grouped into three categories: clinical, radiological, and integrated. In clinical practice, there is no doubt its convenience to predict associated outcome with merely a single radiological and clinical scale. For clinical grading scales, such as the HH, WFNS and GCS, were originally designed to predict treatment and prognosis options for aSAH.

Intriguingly, the SEBES is regarded to be a surrogate marker of early brain injury (EBI) and is used to qualitatively evaluate the degree of cerebral edema. To some degree, the high-grade SEBES may increase the risk for potential DCI after hemorrhage. Meanwhile, the radiological factor, “mFS,” according to quantitative blood volume, is likely to reflect changes the degree of EBI and influence the prediction of DCI. Although it has been shown that higher mFS is an important risk factor to predict DCI (11). In addition, a higher WFNS score was strongly associated with the DCI in patients with aSAH (12). The WFNS score is derived from the GCS score and focuses on symptoms and signs of SAH at admission, reflecting the degree of brain damage. It is widely used in several comprehensive grading systems such as the modified WFNS and VG12 (13). Furthermore, additional intracerebral hematoma has been observed in patients with aSAH and is associated with the development of DCI (14). The underlying mechanism explaining the high incidence of DCI caused by intracerebral hematoma may be that the mass effect of hematoma leads to a long-term and sustained increase in intracranial pressure (15). In theory, the earlier the hematoma is removed, the better the prognosis. Claassen et al. (16) confirmed that lateral ventricular IVH can be an independent predictor of DCI.

The predictive grading of radiological, clinical, and composite scores have been studied before. Unfortunately, the comparative results of various studies are contradictory (3, 10). In a review of the literature, a recent study compared the 3 scores in 423 patients with aSAH indicated that composite grading scores (e.g., HAIR, VG) was not superior to clinical scores (e.g., WFNS, HH), either in predicting unfavorable outcome or cerebral infarction (12, 17, 18). In addition, even radiological scales, such as the mFS, are applied for the prediction of DCI or vasospasm, and ignore the significance of intraparenchymal hematoma or IVH, as mentioned above (19). Among SEBES scores in this study, mFS demonstrated the lowest AUC (0.728) to predict the prognosis of adverse outcomes compared with clinical scores (e.g., WFNS, HH). However, these clinical scores may underestimate risks in patients who are conscious, exhibit no severe nerve defects, and existing IVH or thick subarachnoid blood clots. Subsequently, some researchers have proposed composite scores to overcome the shortcomings of separate radiological and clinical scores (5).

It should be noted that other risk stratification scores often have wide scoring ranges, which may limit their applicability and inconvenience practitioners (20). Different from other risk stratification scores, the web-based dynamic nomogram makes our score easier to use. Unlike the traditional multivariate regression logistic method, LASSO regression analysis was used in this study, which performed well in decreasing multicollinearity between variables and decreasing data dimensionality, which was applied to further multivariable logistic regression by reducing variance and coefficients (21). Then, a related logistic regression model was applied to build a nomogram. In our study specifically, the original AUC was 0.84, while the bias-corrected C-index with 1,000 bootstraps was 0.82, indicating that this model demonstrated exceptional discrimination. The nomogram model can effectively predict the development of DCI better than other prognostic factors.

Therefore, the well-validated prognostic model could accurately predict prognosis according to clinical needs. Individualized treatment based on prognostic model prediction is important to provide recommendations for management. However, the present study had some limitations, the first of which was just two centers, retrospective, observational design, from which our conclusions were drawn. It should be noted that each clinical score was obtained from detailed medical records in which signs and symptoms were carefully documented. More importantly, DCI confirmation and radiological scores were performed by 2 surgeons who were blinded to the design and patient information. To reduce potential deviation, patients who experienced onset of aSAH > 72 h before CT were excluded to assure timing for the assessment of each variable. We also removed patients with severe comorbidities that may have been associated with a cerebrovascular event before the onset of SAH because this could affect clinical judgment. Second, the uneven distribution of patients at different levels may be a general limitation of the risk score. This may be due to the scoring task for all variables are different. Third, although the performance of the EDCI score has been validated, it was not rigorously completed. As such, it will be necessary to test our model using patient outcomes and clinical characteristics at other centers.

## Data availability statement

The original contributions presented in the study are included in the article/supplementary material, further inquiries can be directed to the corresponding authors.

## Ethics statement

The studies involving humans were approved by the Ethics Committee of Renmin Hospital of Wuhan university. The studies were conducted in accordance with the local legislation and institutional requirements. Written informed consent for participation was not required from the participants or the participants’ legal guardians/next of kin in accordance with the national legislation and institutional requirements.

## Author contributions

SC: Writing – original draft, Writing – review & editing. HJ: Writing – original draft, Writing – review & editing. PH: Writing – original draft. XT: Writing – original draft, Writing – review & editing. QC: Writing – original draft, Writing – review & editing.

## Footnotes

^1^ https://www.r-project.org/

## References

[1] Venketasubramanian N Yoon BW Pandian J Navarro JC. Stroke epidemiology in south, east, and south-east asia: a review. J Stroke. (2017) 19:286–94. doi: 10.5853/jos.2017.00234, PMID: 29037005 PMC5647629

[2] Chen X Luo X Hu H Xu Q. NBTI attenuates neuroinflammation and apoptosis partly by ENT1/NLRP3/Bcl2 pathway after subarachnoid hemorrhage in rats. Neuroreport. (2021) 32:1341–8. doi: 10.1097/WNR.0000000000001733, PMID: 34718248 PMC8560159

[3] Ahn SH Savarraj JP Pervez M Jones W Park J Jeon SB. The subarachnoid hemorrhage early brain edema score predicts delayed cerebral ischemia and clinical outcomes. Neurosurgery. (2018) 83:137–45. doi: 10.1093/neuros/nyx364, PMID: 28973675

[4] Rajajee V. Grading scales in subarachnoid hemorrhage—many options, but do we have a winner? Eur J Neurol. (2018) 25:207–8. doi: 10.1111/ene.13516, PMID: 29115698

[5] de Oliveira MA Jaja BN Germans MR Yan H Qian W Kouzmina E. The vasograde: a simple grading scale for prediction of delayed cerebral ischemia after subarachnoid hemorrhage. Stroke. (2015) 46:1826–31. doi: 10.1161/STROKEAHA.115.008728 25977276

[6] Fang YJ Mei SH Lu JN Chen YK Chai ZH Dong X. New risk score of the early period after spontaneous subarachnoid hemorrhage: for the prediction of delayed cerebral ischemia. CNS Neurosci Ther. (2019) 25:1173–81. doi: 10.1111/cns.13202, PMID: 31407513 PMC6776741

[7] Fang Y Lu J Zheng J Wu H Araujo C Reis C. Comparison of aneurysmal subarachnoid hemorrhage grading scores in patients with aneurysm clipping and coiling. Sci Rep. (2020) 10:9199. doi: 10.1038/s41598-020-66160-0, PMID: 32513925 PMC7280262

[8] Frontera JA Claassen J Schmidt JM Wartenberg KE Temes R Connolly EJ. Prediction of symptomatic vasospasm after subarachnoid hemorrhage: the modified fisher scale. Neurosurgery. (2006) 59:21–7. doi: 10.1227/01.neu.0000243277.86222.6c, PMID: 16823296

[9] Li X Zeng L Lu X Chen K Yu M Wang B. Early brain injury and neuroprotective treatment after aneurysmal subarachnoid hemorrhage: a literature review. Brain Sci. (2023) 13:1083. doi: 10.3390/brainsci13071083, PMID: 37509013 PMC10376973

[10] Maragkos GA Enriquez-Marulanda A Salem MM Ascanio LC Chida K Gupta R. Proposal of a grading system for predicting discharge mortality and functional outcome in patients with aneurysmal subarachnoid hemorrhage. World Neurosurg. (2019) 121:e500–10. doi: 10.1016/j.wneu.2018.09.148, PMID: 30268551

[11] Rinaldo L Rabinstein AA Lanzino G. Increased body mass index associated with reduced risk of delayed cerebral ischemia and subsequent infarction after aneurysmal subarachnoid hemorrhage. Neurosurgery. (2019) 84:1035–42. doi: 10.1093/neuros/nyy104, PMID: 29659999

[12] Neidert MC Maldaner N Stienen MN Roethlisberger M Zumofen DW D’Alonzo D. The barrow neurological institute grading scale as a predictor for delayed cerebral ischemia and outcome after aneurysmal subarachnoid hemorrhage: data from a nationwide patient registry (swiss sos). Neurosurgery. (2018) 83:1286–93. doi: 10.1093/neuros/nyx609, PMID: 29351673

[13] Zou Y Zhang C Ge H Li H Fang X Zhong J. Comparison of epidemiological and clinical features between two chronological cohorts of patients with intracerebral hemorrhage. J Clin Neurosci. (2020) 72:169–73. doi: 10.1016/j.jocn.2019.12.031, PMID: 31911108

[14] Liu H Xu Q Li A. Nomogram for predicting delayed cerebral ischemia after aneurysmal subarachnoid hemorrhage in the chinese population. J Stroke Cerebrovasc Dis. (2020) 29:105005. doi: 10.1016/j.jstrokecerebrovasdis.2020.105005, PMID: 32807421

[15] Sehba FA Pluta RM Zhang JH. Metamorphosis of subarachnoid hemorrhage research: from delayed vasospasm to early brain injury. Mol Neurobiol. (2011) 43:27–40. doi: 10.1007/s12035-010-8155-z, PMID: 21161614 PMC3023855

[16] Jin X Wang S Zhang C Yang S Lou L Xu S. Development and external validation of a nomogram for predicting postoperative pneumonia in aneurysmal subarachnoid hemorrhage. Front Neurol. (2023) 14:1251570. doi: 10.3389/fneur.2023.1251570, PMID: 37745673 PMC10513064

[17] Dengler NF Sommerfeld J Diesing D Vajkoczy P Wolf S. Prediction of cerebral infarction and patient outcome in aneurysmal subarachnoid hemorrhage: comparison of new and established radiographic, clinical and combined scores. Eur J Neurol. (2018) 25:111–9. doi: 10.1111/ene.13471, PMID: 28940973

[18] Akkol S. The prediction of live weight of hair goats through penalized regression methods: lasso and adaptive lasso. Arch Anim Breed. (2018) 61:451–8. doi: 10.5194/aab-61-451-2018, PMID: 32175452 PMC7065407

[19] Ko SB Choi HA Helbok R Schmidt JM Badjatia N Claassen J. Quantitative analysis of hemorrhage clearance and delayed cerebral ischemia after subarachnoid hemorrhage. J Neurointerv Surg. (2016) 8:923–6. doi: 10.1136/neurintsurg-2015-011903 26276078

[20] Lee H Perry JJ English SW Alkherayf F Joseph J Nobile S. Clinical prediction of delayed cerebral ischemia in aneurysmal subarachnoid hemorrhage. J Neurosurg. (2018) 130:1–8. doi: 10.3171/2018.1.JNS172715 29882700

[21] Russin JJ Montagne A D’Amore F He S Shiroishi MS Rennert RC. Permeability imaging as a predictor of delayed cerebral ischemia after aneurysmal subarachnoid hemorrhage. J Cereb Blood Flow Metab. (2018) 38:973–9. doi: 10.1177/0271678X18768670, PMID: 29611451 PMC5998996

